# An Advanced Combinatorial System from *Vitis vinifera* Leaves and Propolis Enhances Antioxidants’ Skin Delivery and Fibroblasts Functionality

**DOI:** 10.3390/ph17121610

**Published:** 2024-11-29

**Authors:** Sophia Athanasopoulou, Eleni Spanidi, Eleni Panagiotidou, Andrea Cavagnino, Anaïs Bobier, Konstantinos Gardikis

**Affiliations:** 1 Research and Development Department, APIVITA SA, Industrial Park Markopoulo Mesogaias, 19003 Athens, Greece; sofia.athanasopoulou@puig.com (S.A.); eleni.spanidi@puig.com (E.S.); eleni.panagiotidou@puig.com (E.P.); 2 OxiProteomics SAS, 2 Rue Antoine Etex, 94000 Creteil, France; 3 Department of Pharmacy, Frederick University, Nicosia 1036, Cyprus

**Keywords:** polyphenols, collagen, elastin, skin delivery system

## Abstract

**Background/Objectives**: Vine leaves are a bulky by-product that are disposed of and treated as waste in the wine production process. In the present study polyphenols from vine leaves were extracted and simultaneously encapsulated in a new delivery system consisting of liposomes and cyclodextrins. This system was further combined with propolis polyphenols encapsulated in cyclodextrins, resulting in a colloidal suspension for the release of antioxidants in a time-controlled way, the rate of which depends on the ratio of the materials. The result is a raw material that exhibits antioxidant and ECM protective effects when administered in skin fibroblasts (NHDFs). **Methods**: The antioxidant and ECM promoting efficacy of the produced raw material was assessed by the Folin–Ciocalteu method, DPPH assay, and in cellulo assays in fibroblasts, such as the cell viability assay, scratch assay, cell migration assay, gene expression analysis, and immunofluorescence analysis, for the detection, visualization, and quantification of collagen-I, collagen-IIIa, and elastin signals and collagenase assay. **Results**: Treatment of NHDFs with the combinatorial delivery system promoted collagen and elastin synthesis and deposition in normal conditions and, upon induced external stress, as assessed by in vitro transcriptomic and proteomic analysis. A significant inhibition of collagenase was also observed, suggesting a multitargeted efficacy of the active ingredients also by preventing collagen degradation. **Conclusions**: Therefore, this liposome–cyclodextrin encapsulated polyphenol complex represents a novel bioactive ingredient with promising skin applications.

## 1. Introduction

Skin aging is a complex process that involves various biological mechanisms, including oxidative stress, inflammation, and degradation of the extracellular matrix (ECM) components. Age-dependent deterioration of the ECM involves alterations in collagen turnover and decreased levels of elastin—two essential proteins for healthy skin tissue. In young skin, collagen provides structure and support to the skin, while elastin helps it to stay flexible and elastic. As skin ages, the production of collagen and elastin by fibroblasts naturally declines, leading to wrinkles, sagging skin, and other signs of aging. Collagen crosslinks stabilize, while collagen bundles become disorganized. Polyphenols are a group of natural compounds widely found in plants and have been reported to exhibit strong antioxidant activity [1]. Polyphenols deriving from natural sources, have been shown to help reverse this decline by stimulating the production of functional collagen and elastin in skin cells [2,3,4,5].

Vine leaves are a bulky by-product which is generated during winemaking and viticulture processes, often considered as agricultural waste, and discarded or burned, leading to environmental pollution. Their applications to date have been limited, at best, to a small number of edible products and at worst to the creation of a soil enhancer. However, recent research has shed light on the potential of upcycling waste from vine leaves into valuable cosmeceutical ingredients [6,7]. Grapes and grape products, as sources of bioactive chemicals applied in cosmetic manufacture, constitute an environmentally friendly alternative for generated residues. The active ingredients from these sources are potentially very valuable to the cosmetics industry, which is always in search of new and sustainable sources for effective raw materials. Vine leaves are rich in various bioactive compounds, including polyphenols, flavonoids, and organic acids, which are known for their antioxidant, anti-inflammatory, and anti-aging properties [8]. Flavonoids are a type of polyphenol that have been shown to possess anti-inflammatory properties and protect the skin from UV radiation-induced damage [9,10]. Organic acids, such as tartaric acid and malic acid, are also present in vine leaves, and have been reported to exhibit exfoliating and brightening effects on the skin [11]. These bioactive compounds make waste from vine leaves a promising source of cosmeceutical ingredients.

Polyphenols are the most abundant and the most studied class of bioactive compounds present in *Vitis vinifera* leaves. They represent secondary metabolites and include phenolic acids, coumarins, flavonoids, stilbenes, and lignans [8]. Previous studies from our lab on vine leaf extracts from the area of Santorini, Greece, have indicated significant transcriptional and epigenetic effect on UVA stressed fibroblasts. The specific extract was shown to activate two genes with important roles in the anti-aging and ECM organization processes, namely SIRT-1 and HSP-47, via their transcriptional upregulation, which has been shown to be mechanistically linked with an observed demethylation of their promoters.

Propolis is another natural product that demonstrates important antioxidant, anti-aging and anti-inflammatory activities [12,13,14]. Raw propolis, a natural resinous substance produced by honeybees (*Apis mellifera* L.), results from the combination of their saliva containing enzymes, beeswax, and resins from plant sources. Its main constituents include 50–60% resins, 30–40% waxes and fatty acids, 5–10% essential oils, approximately 5% pollen, and 5% other compounds, such as amino acids and vitamins. The chemical composition, colors, and biological activities of propolis vary based on bee race, geographical origin, and the specific plant or tree species used by bees for collecting pollen and exudates, as well as the sampling season [15,16,17,18]. According to a plethora of studies, the main secondary metabolites in propolis are phenolic substances, especially flavonoids, belonging to different sub-classes such as flavanones, flavones, flavonols, and dihydroflavonols, which constitute more than 50% of the propolis weight. Other phenolic compounds found in abundance in propolis are hydroquinones, caffeic acids and related esters, and phenolic aldehydes [19].

In recent years, the interest in the potential use of propolis polyphenols in cosmeceutical applications, including skincare products has increased, due to their beneficial effects on skin health and appearance.

Several studies have investigated the anti-aging effects of propolis polyphenols in cosmeceutical applications [20,21]. For instance, a study by González-Masís et al. (2020) found that propolis polyphenols increased cell metabolic activity and cell proliferation in human skin fibroblasts, mechanisms that are responsible for synthesizing ECM components, such as collagen and elastin [22]. Another study by Franchin et al. (2016) evaluated in vivo, in mammalian model organisms the effects of isoflavonoids of propolis, in acute and chronic inflammation and found a potential anti-inflammatory activity that could alleviate skin tissue aging due to chronic inflammation [23,24].

Complementary to the anti-aging effect of propolis, several studies have demonstrated the antioxidant effects of propolis polyphenols in cosmeceutical applications. For example, a study by Lagouri et al. (2014) found that propolis polyphenols extracted from Greek propolis exhibited strong antioxidant activity, as evidenced by their ability to scavenge reactive oxygen species (ROS) [25]. Another in vitro study has demonstrated the antioxidant activity of propolis polyphenols by inhibiting lipid peroxidation in human skin cells [26]. The antioxidant effects of a propolis-based cream in human volunteers were evaluated by Diniz et al. (2019) who found that the cream significantly reduced biomarkers of oxidative stress cell damage in healthy humans, with increased antioxidant enzymatic capacity, especially of superoxide dismutase (SOD) [27]. Previous studies have identified that propolis extracts collected from different geographical areas in Greece demonstrate significant differentiation on their chromatographic profile and concentration of bioactive components, with this diversity also being reflected in their biological activity [28,29]. For instance, samples originating from Central Greece are characterized as very rich in flavonoids, compared to samples from other regions of Greece, and show high antioxidant and anti-collagenase activity [30].

Studies from our lab on a specific propolis extract from Olympus Mountain (Central Greece) have demonstrated that it confers significant protection against the cytotoxic effects of UVB radiation by remarkably reducing DNA and protein oxidation damage levels in human immortalized keratinocytes (HaCaT). It also demonstrated significant anti-aging efficacy, as it decreased histological damage and also decreased the induced expression of certain metalloproteinases (MMPs) following exposure to UVB in a reconstituted skin model [9].

To address a complex issue, such as UV-induced dermal cell damage, our target was to develop a combinatorial delivery system incorporating polyphenols from different sources and of different types, called the vine propolis delivery system (VPDS), aiming at specific molecular targets. Propolis and *Vitis vinifera* leaves were chosen as polyphenol sources due to the high concentration of different polyphenols they contain, and due to the significant efficacy of those specific extracts in mechanisms that support skin cell functionality. Our analytical results were in line with the literature [31,32].

More specifically, polyphenols from vine leaves were extracted and simultaneously encapsulated in a new combinatorial system for cosmeceutical use, consisting of liposomes and cyclodextrins. In parallel, polyphenols from propolis collected from Mount Olympus, Greece, were encapsulated in the system resulting in a colloidal suspension that releases polyphenols in a time-controlled way.

In the present study, which presents the results of research concerning an innovative approach to natural anti-aging biomaterials, we focused on the antioxidant activity of the combinatorial system of vine leaves and propolis polyphenols and its potential to synergistically increase the functionality of fibroblasts in protein levels, by stimulating collagen and elastin protein production, along with its effect in cell proliferation, cell migration, and the transcriptional regulation of genes implicated in cellular growth, cell adhesion and contractility.

## 2. Results

### 2.1. Experimental Results

#### 2.1.1. Phytochemical Assays

The encapsulation efficiency of polyphenols in the VPDS 91% ± 4% when the initial materials contain total polyphenol compounds exceeded 16 µg GA/mL, as described in the Materials and Methods section.

Table 1 depicts the mean hydrodynamic diameter, polydispersity index, and ζ-potential of the system at day 1, and after 1 month at various temperatures. The values remain practically stable except for the temperature of 38 °C, where there is a statistically significant increase in particle size, possibly attributed to particle aggregation.

The initial concentration of total phenols was 709 ± 22 μg GA/mL, while the antioxidant capacity was 5.23 ± 2.39 mM Trolox equivalents. Over the course of 24 weeks, both the antioxidant capacity and total polyphenols remained stable without significant fluctuations (Table 2).

**Figure 1:**
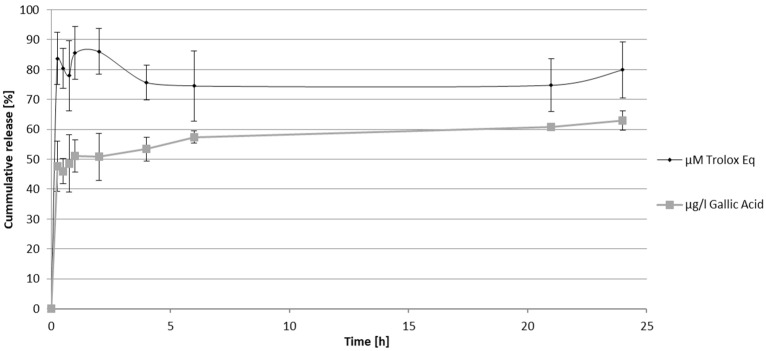
In vitro release profile of vine leaves and propolis combinational extract in cyclodextrins measuring antioxidant activity against DPPH free radical (μΜ Trolox Eq.) (± SD) and total phenolic content-TPC (μg/L gallic acid) (± SD).

The values of phytochemical parameters, such as pH value, refractive index, and density, were stable at different temperatures and time points for 24 weeks. The in vitro controlled release of the VPDS system was studied using only cyclodextrins and prior to the addition of the liposome (Figure 1).

As shown in Figure 1, about 80% of the antioxidant activity is released in the first 15 min and, at the same time, about 50% of the total phenolic compounds are released.

**Figure 2:**
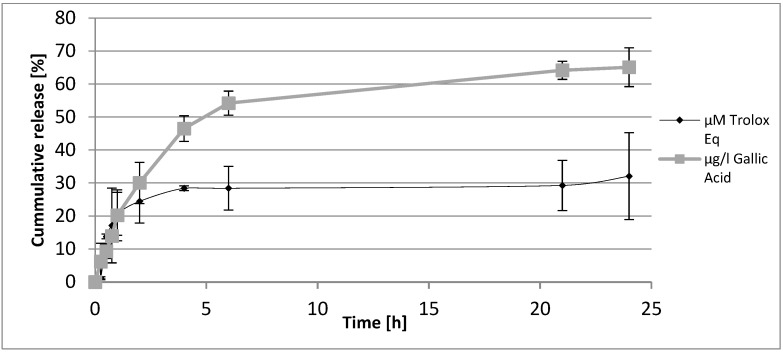
In vitro release of antioxidant activity with free radical DPPH (μΜ Trolox Eq.) (±SD) and total phenolic content -TPC (μg/L gallic acid) (±SD) of VPDS for time 0 to 24 h.

The in vitro release profile of VPDS system as a function of time is presented below in Figure 2. The release of the antioxidant activity reaches practically a plateau at 4 h and the content of the total phenolic compounds are reached at 6 h.

#### 2.1.2. Cell Viability and Migration Assay

**Figure 3:**
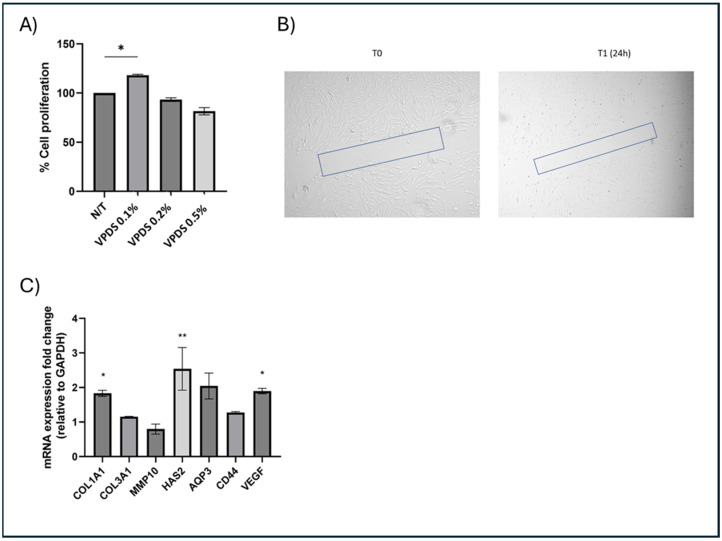
(**A**). Cell viability assay showing that VPDS 0.1% *v*/*v* increased cell metabolism and proliferation by +20%; (**B**). Cell migration assay. Representative images from the wound healing assay of NHDF cell cultures treated with the active ingredients demonstrated that cell invasion into the cell-free region (outlined) after 24 h of treatment with VPDS 0.1% *v*/*v* (decreasing the cell free area by 33%); (**C**). Transcriptional regulation of key genes of fibroblasts’ functionality showing that VPDS 0.1% *v*/*v* increased the expression of key genes: +80% for Col1A1, +150% for HAS2, and +90% for VEGF. (* *p*-value < 0.05,** *p*-value < 0.01).

To assess the effect of the active ingredients on cell viability, an MTT Assay using three different concentrations of VPDS (0.1% *v*/*v*, 0.2% *v*/*v* and 0.5% *v*/*v*) was performed in NHDFs. An increase in cell metabolism and proliferation by 20% was demonstrated with the concentration of active ingredients at 0.1% *v*/*v*. This effect was not observed when the concentration was doubled or 5 times higher (Figure 3A).

Following the cell viability assay that determined the optimal concentration of the active, we performed a cell migration assay (scratch assay) in a confluent monolayer of NHDFs to assess the potential wound healing action of the active ingredients. The in vitro cell migration assay (scratch assay) that was conducted in NHDFs demonstrated a significant closure (−33%) of the “wound” after 24 h of treatment with VPDS 0.1% *v*/*v* (Figure 3B).

#### 2.1.3. Transcriptomic Assays

To further examine the observed increase in cell proliferation and wound healing, we tested potential transcriptional changes in key genes of fibroblast functionality, related with ECM remodeling, hyaluronan synthesis, and a major orchestrator of tissue repair and wound healing, VEGF. A real time PCR analysis demonstrated that VPDS 0.1% *v*/*v* increased the expression of key genes of fibroblasts’ functionality: +80% for Col1A1; +150% for hyaluronan synthase-2 (HAS2); and +90% for VEGF (Figure 3C).

#### 2.1.4. Immunofluorescence Assays

The efficacy of VPDS 0.1% *v*/*v* was additionally tested upon exposure of fibroblasts to a combination of stressors, namely UVA irradiation and the addition of particulate matter (ERMCZ100 PM10-like—PAHs; 1 μg/cm^2^), mimicking the condition of stress from urban pollution.

**Figure 4:**
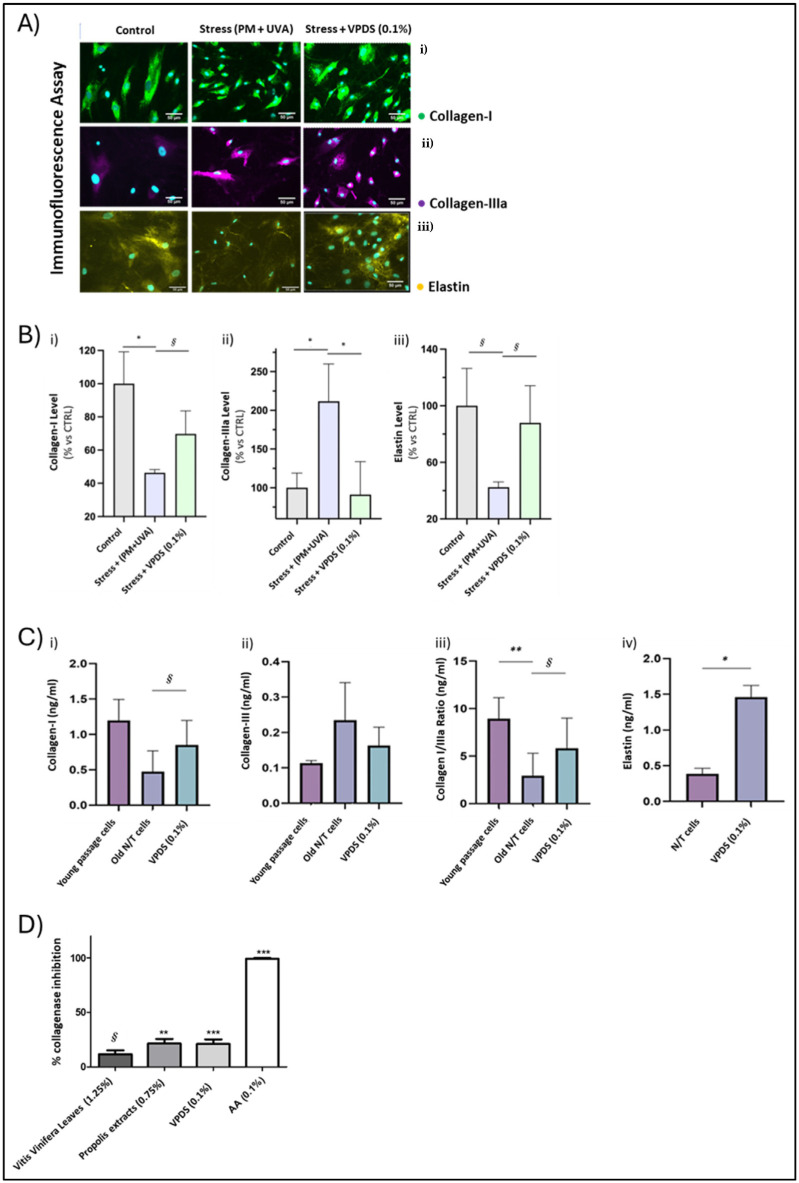
(**A**) (**i**) In situ visualization of Collagen-I (in green). The specific signal of collagen-I immunofluorescence detection is visualized in green and superposed to cell nuclei (DAPI, in cyan). (**ii**) In situ visualization of collagen-IIIa (in purple). The specific signal of collagen-IIIa immunofluorescence detection is visualized in purple and superposed to cell nuclei (DAPI, in cyan). (**iii**) In situ visualization of elastin (in yellow). The specific signal of elastin immunofluorescence detection is visualized in yellow and superposed to cell nuclei (DAPI, in cyan). (**B**) (**i**) Graph bar representation of the quantification of collagen-I by detection of specific signal normalized by the number of cells and on the control group * *p* < 0.05 *§ p* < 0.1—*t*-test for binary comparisons versus stress group (unpaired, two-ways). (**ii**) Graph bar representation of the quantification of collagen-IIIa by detection of specific signal normalized by the number of cells and on the control group * *p* < 0.05—*t*-test for binary comparisons versus stress group (unpaired, two-ways). (**iii**) Graph bar representation of the quantification of elastin by detection of specific signal normalized by the number of cells and on the control group *§ p* < 0.1—*t*-test for binary comparisons versus stress group (unpaired, two-ways). (**C**) (**i**) Graph bar representation of the quantification of collagen-I by detection of protein level in NDHFs *§ p* < 0.1—*t*-tests for comparison of young passage fibroblasts untreated, old passage untreated and old passage treated. (**ii**) Graph bar representation of the quantification of collagen-IIIa by detection of protein level in NDHFs *§ p* < 0.1—*t*-tests for comparison of young passage fibroblasts untreated, old passage untreated and old passage treated. (**iii**) Graph bar representation of the ratio of collagen-I/-IIIa in young passage cells and upon replicative senescence of treated and untreated NHDFs. (**iv**) Graph bar representation of the quantification of elastin by detection of protein level in NDHFs * *p* < 0.05 *t*-tests for comparison old passage fibroblasts untreated and treated. (**D**) Inhibition of collagenase. Mean (±SEM, N = 4), *** *p* < 0.001 ** *p* < 0.01 *§ p* < 0.1—one-way ANOVA (Dunnett’s multiple comparisons test).

VPDS 0.1% *v*/*v* counteracted the changes induced by the UVA/pollution stress in dermal fibroblasts, restoring the levels of collagen and elastin. More specifically, in situ analyses of collagen-I, collagen-IIIa, and elastin were performed by epi-fluorescence microscopy via specific immunofluorescence detection. As shown in Figure 4, the presence of the active ingredient cancelled out the effects of stress exposure.

The quantification of collagen-I levels was carried out by integrating the intensity of the specific signal over the surface of evaluation, normalized by the number of cells. Three images per condition were analyzed and the results are reported as histograms (Figure 4A(i),B(i)). The levels of collagen-I decreased upon pollution exposure. The presence of VPDS 0.1% *v*/*v* contrasted with the stress-induced decrease of collagen-I.

Quantification of the collagen-IIIa levels was carried out by integrating the intensity of the specific signal over the surface of evaluation, normalized by the number of cells. Three images per condition were analyzed, and the results are reported as histograms (Figure 4A(ii),4B(ii)). The levels of collagen-IIIa increased upon pollution exposure. The presence of VPDS 0.1% *v*/*v* counteracted the stress-induced increase of collagen-IIIa.

Similarly, for elastin levels, their quantification was carried out by integrating the intensity of the specific signal over the surface of evaluation, normalized by the number of cells. Three images per condition were analyzed and the results are reported as histograms (Figure 4A(iii),B(iii)).

A significant decrease of the collagen-I levels was observed in NHDFs, where pollution stress was applied in comparison with unstressed NHDFs. For collagen-IIIa, a significant increase of its levels was detected upon stress, in comparison with both unstressed NHDFs, but also with cells treated with the active ingredients for 24 h, where the increase was efficiently counteracted.

#### 2.1.5. ELISA-Enzymatic Assays

After having confirmed that the active ingredients counteracted the changes induced by the UVA/pollution stress in dermal fibroblasts by restoring levels of collagen and elastin, we further investigated its effects in collagen and elastin production in late passage fibroblasts. A statistically significant increase in elastin levels was confirmed in treated NHDFs with a 4-fold change, compared to untreated cells. Moreover, we measured the levels of the two types of collagen and their ratio in untreated and treated with the active ingredients in late passage NHDFs, in comparison with the levels from young passage NHDFs, as the immunofluorescence experiments had shown an increase of collagen-IIIa upon exposure to pollution stress and a decrease of collagen-I levels. A statistically significant difference was observed between the ratio of young passage dermal fibroblasts and untreated late passage fibroblasts for the two types of collagen, with the ratio of the latter being 3-fold lower than the ratio of young passage fibroblasts.

Additionally, we tested the inhibitory effect of the VPDS, along with its different components (*Vitis vinifera* leaves and propolis extracts) in the enzyme collagenase. The complex showed a statistically significant inhibition of collagenase at the same rate with the propolis extract, leading to the assumption that the inhibitory action is mainly attributable to the latter.

## 3. Discussion

Combining vine leaves polyphenols with propolis polyphenols may have several potential benefits due to their individual and synergistic effects. We here focused on the combination’s beneficial effects in dermal fibroblast’s functionality for potential use as a cosmeceutical with anti-aging and antioxidant properties.

Using a high diversity of polyphenols has been previously shown to increase the potential biological benefits of those molecules [4], and our screening in vitro results proved this assumption).

To overcome the issue of polyphenol degradation over time, vine leaves and propolis polyphenols were encapsulated in two distinct delivery systems: liposomes/cyclodextrins and cyclodextrins, respectively [33,34]. The reasoning behind the choice of those materials was initially the already demonstrated ability of combinatorial liposome/cyclodextrin systems to achieve controlled release of encapsulated substances. The use of the combination of liposomes and cyclodextrins is based on the concept of advanced drug delivery nanosystems [35]. In such systems, materials of a different nature are used aiming to modulate the release rate of encapsulated molecules, as well as to increase entrapment efficiency and low leakage of drugs. Specifically on the interaction of cyclodextrins with liposomes, several successful attempts have been performed [33,34,36]. Such drug-in-cyclodextrin-in-liposome (DCL) systems are based on the physicochemical interactions between cyclodextrins and phospholipids resulting in creating membranes of altered fluidity that can prove beneficial for controlled delivery of bioactive molecules [37].

For propolis polyphenols, a propolis–cyclodextrin complex was designed, achieving an almost instant release of polyphenols, polyphenol stability, as well as solubility in highly polar solvents.

This technological platform that can deliver a wide diversity of polyphenols to the human skin in a time-controlled way was assessed against various factors of skin aging.

Aging skin is known to have an impaired barrier function, which leads to a dry appearance and increased sensitivity to external aggressors, increasing the risk for skin problems [38]. The principal changes in aging skin, aside from the uneven texture and pigmentation of the epidermis, take place at the level of the dermal connective tissue and primarily manifest as the loss of mature collagen and modifications to the elastic network [39]. Elastases and matrix metalloproteinases (MMPs) are two of the enzymes that break down ECM. These endopeptidases play significant roles in several physiological processes, including tissue repair and remodeling, cell migration and differentiation, or wound healing [40,41]. They are responsible for the turnover of several ECM components, including all types of collagen and elastin, while increased levels of their enzymatic activities have been associated with a variety of pathological conditions and adverse effects, including accelerated skin aging.

It is well recognized that phenolic chemicals, such as the primary phenolics found in red grapes called anthocyanins, not only have well-known antioxidant characteristics but have the power to directly inhibit skin-aging enzymes, such as collagenase (MMP-1). In this regard, it was expected and finally proven that these bioactive compounds encountered in the winery residues used, let alone in their combination with propolis polyphenols, exhibited potential ECM protecting properties.

To assess the bioactivity of this in-house produced new combinatorial polyphenol release system, we first examined the effects of different concentrations of VPDS on cellular proliferation and metabolic activity via an MTT assay. Given the important observed increase in cell viability and metabolism of NHDFs treated with VPDS in the concentration of 0.1%, we assessed the molecular activity of the active ingredients by targeting a panel of genes representative of ECM structural proteins, in association with regulatory genes involved in pathways that modulate growth factors, cell adhesion, and contractility.

We observed an enhanced expression of collagen-I, one of the most important providers of structural support to the ECM and a well-known activator of dermal fibroblasts [42,43]. The levels of the ECM protein elastin also increased upon treatment with VPDS. This outcome, in association with the upregulated expression of the Col1A1 gene is indicative of a regulatory role of the system in normal ECM remodeling. Interestingly, the increase in elastin protein levels is confirmed not only upon treatment of NHDFs exposed in UV and pollution stress, but in fibroblasts that had undergone replicative senescence.

More specifically, a statistically significant increase in elastin levels was confirmed in old passage NHDFs treated with VPDS. Moreover, we observed a decrease in collagen-I protein levels accompanied by a simultaneous increase in collagen-IIIa in NHDFs that had undergone pollution stress (exposure to UVA and particulate matter). Interestingly, we show that collagen-I and collagen-IIIa protein levels were reversed after 24 h treatment with VPDS. Thus, we evaluated and compared the ratio of the protein levels of collagen-I and collagen IIIa (collagen type I/III) in untreated and treated late passage NHDFs with the ratio of untreated young passage NHDFs. A statistically significant difference was observed between the collagen ratio of young passage dermal fibroblasts and untreated late passage fibroblasts. Interestingly, in a translational approach of these findings, a similar observation in the ratio of the two types of collagen has also been reported in vivo and more specifically in the skin of patients where skin deformation type of facial aging was assessed. Early development of ptosis has been more rapid in patients with a reduced collagen type I/III ratio. Histological examination of these patients indicated the immaturity of connective tissue, suggesting a prognostic value of the collagen-I/IIIa ratio [44].

Fibrillar collagen-IIIa is found in abundance in the skin of the fetus, while less is found in adult skin. Moreover, it can be found in fibers along with type I collagen fibrils in the reticular organs and blood vessel walls [45]. Collagen-IIIa is abundant in hollow organs, such as vocal cords, but it also interacts with platelets during blood coagulation (through specific glycoproteins and non-integrin receptors) and is a crucial signaling molecule in tissue regeneration (by taking part in cellular adhesion, migration, proliferation, and differentiation through interaction with receptors on the cell surface, including integrins) [46]. In the transcriptional study, the expression of the Col3A1 gene did not present upregulation, suggesting that in normal conditions collagen type IIIa is beneficial when expressed in steady levels.

A significant upregulation of HAS2 expression was also demonstrated, suggesting the implication of the ingredient in hyaluronic acid (HA) synthesis. HA is a basic linear polymer that results in a massive hydrophilic molecule that imparts a significant amount of moisture and supports the turgor and flexibility of healthy skin. Yet HA serves vital metabolic and physiological purposes that were previously largely underappreciated, in addition to aesthetic ones. By its interaction with the important cell-surface receptor CD44, extracellular HA regulates the fibroblasts’ physiology. Via CD44 interactions with the cytoskeleton, EGF, and TGF receptors, this relationship promotes intracellular signaling for cellular proliferation both directly and indirectly [47]. Stress conditions, either UVB exposure or serum starvation, have been shown to reduce HAS2 expression in human primary skin fibroblasts, resulting in an increased susceptibility to stress-induced apoptosis [48]. Increase in HAS-2 expression, therefore, may contribute to efficient HA generation for the development of a pericellular HA coat and supporting and stimulating ECM molecule production [47]. An increasing trend was also demonstrated in both the CD44 gene that encodes the receptor of HA, along with the Aquaporin-3 (AQP-3) gene. HA is a natural regulator of AQP-3, a protein responsible for transmembrane water transport in human skin, with an important role in the epithelial barrier structure; thus, it is possible that HAS2 upregulation may trigger in a latter phase an increase also in this protein, as they have a synergetic function in the skin ECM [49].

Interestingly, and in accordance with the wound healing effect of the bioactive ingredient, an increase in the expression of VEGF was also detected. As the latter has been shown to orchestrate the expression of growth factor genes and cell migration, its upregulation is coherent with previous studies that have emphasized the role of known vine polyphenols also in wound healing in vitro and in model organisms [50]. VEGF is also a factor that, apart from being a key mediator in vascular remodeling, has also been demonstrated to promote specific ECM synthesis and fibroblast activation [51]. Moreover, regarding wound healing processes, collagen-I, which plays a crucial function in enhancing connective tissue by maintaining tissue health and providing an ECM outline for cellular adhesion and migration, is also involved. Collagen’s role in wound healing is to attract fibroblasts and make the deposition of newly formed collagen easier [23]. Massive MMPs are produced by chronic wounds, which hinder the normal healing process [52]. The ECM’s excessive MMPs are pickled and arrested by collagen. Hence the extract’s induction of the relative expression of type I collagen can also support wound healing by preventing chronic inflammation [52].

In addition to the demonstrated upregulation in the expression of collagen-I, a significant inhibition of collagenase was also observed, suggesting a multitargeted efficacy of the active both by enhancing the formation of new collagen and by preventing its degradation in the ECM.

Overall, our obtained results demonstrate that the innovative combinatorial system of polyphenols from vine leaves encapsulated in cyclodextrins and liposomes along with propolis polyphenols in cyclodextrins shows significant effects in the functionalities of dermal fibroblasts for an effective ECM remodeling.

Τhese results are in line with the DPPH assay that demonstrated significant antioxidant activity, which was furthermore stable after accelerated aging for one month.

## 4. Materials and Methods

### 4.1. Preparation of Combinatorial Delivery System

To produce the VPDS, *Vitis vinifera* leaves cultivated on Santorini Island in Greece were used at a concentration of 1.5% (*w*/*w*). Additionally, organic certified raw propolis sourced from Greek cultivation, specifically from the Olympus Mountain region (30% *w*/*w*), was incorporated. To optimize encapsulation efficiency, the raw materials—*Vitis vinifera* leaves and raw propolis—should contain total polyphenol compounds exceeding 16 µg GA/mL. This involves dissolving 5% (*v*/*w*) of the raw materials in methanol (PanReac, AppliChem, Darmstadt, Germany) and measuring the resulting solution using a spectrophotometer via the Folin–Ciocalteu method. As solvents, ultrapure water produced at APIVITA with a Reverse Osmosis System and 1.3 propanediol (Connect Chemicals, Vimercate (MB), Italy) in a 60/40 ratio were used. Additionally, 6% (*w*/*w*) hydroxypropyl-β-cyclodextrin (Gangwal Chemicals Pvt. Ltd., Mumbai, India) was dispersed in the solvent system. After seven days, the resulting mixture underwent filtration using a 25 µm nylon bag. Intense stirring at 3000 rpm facilitated the addition of 3% *w*/*w* liposomes (Pro-Lipo™ Neo), which contained propanediol (75.0% *w*/*w*), lecithin (25.0% *w*/*w*), tocopherol (0.25% *w*/*w*), and Helianthus annuus (sunflower) seed Oil (0.15% *w*/*w*). These liposomes were provided by *Generex pharmacist* Pvt. Ltd., Mumbai, India/Lucas Meyer Cosmetics, Massy, France. Following three days of storage at 6 °C, the mixture underwent further filtration through a 0.22 µm Millipore membrane filter and aliquots were stored at 25 °C, 6 °C and 38 °C.

### 4.2. Physicochemical Characterization of VPDS

The VPDS was characterized as described by Spanidi et al. (2021) [33]. The hydrodynamic diameter of the CRPP system was measured by light scattering. Then, 100 μL of the liposomal suspension was diluted 30-fold immediately after preparation or after reconstitution and z-average mean and ζ-potential of the formulations were measured. Samples were scattered (633 nm) at 90° angle, and measurements were made at 25 °C in a photon correlation spectrometer (Zetasizer Pro, Malvern Instruments, Malvern, UK).

### 4.3. VPDS Encapsulation Efficiency

The encapsulation efficiency was calculated by the equation:

EE = entrapped polyphenols/initial polyphenols × 100

Εntrapped polyphenols is the total phenolic content measured in the final system, as described in the TPC section. Initial polyphenols refer to the total phenolic content of the initial materials when dissolved in methanol, as described by Spanidi et al. [33].

### 4.4. Total Phenolic Content (TPC)

The TPC was determined using the Folin-Ciocalteu method according to the method by Arnous et al. [53].

### 4.5. Antioxidant Activity by 2,2-Diphenylpicrylhydrazyl (DPPH)

The antioxidant activity of the colloidal systems was determined by using 2,2-diphenylpicrylhydrazyl (DPPH) free radical as described by Brand-Williams et al. (1995) [54].

### 4.6. In Vitro Release Studies of VPDS

The release rate of the polyphenols of VPDS was measured in a buffer solution at pH 7.2 at 37 °C. An in vitro study of the release of the polyphenols of the extract was performed by using dialysis sacks. A total of 1 mL of the VPDS was placed in the dialysis sacks of a MWCO = 1000 (Pur-A-Lyzer, Midi Dialysis Kit, Sigma-Aldrich, St. Louis, MO, USA). The sack was placed in a buffer pH = 7.2 (CARLO ERBA Reagents, Val-de-Reuil, France) which was constantly stirred with a magnetic bar and thermostated at 37 °C throughout the experiments (hot plate magnetic stirrer, IKA^®^ Werke GmbH & Co., Staufen im Breisgau, Germany). At specific time points (15′, 30′, 45′, and 1, 2, 4, 6, 21, and 24 h), samples were taken, and their polyphenol concentration and antioxidant activity were measured. The buffer quantity removed is replaced by buffer pH = 7.2 at temperature of 37 °C to maintain the sink conditions.

### 4.7. MTT Cell Viability Assay

Cell viability was determined using an MTT colorimetric assay kit Vybrant^®^, Thermo Fisher Scientific, Waltham, MA, USA), following the manufacturer’s protocol.

### 4.8. Cell Migration Assay

Dermal fibroblasts’ monolayer was scraped with a p200 pipet tip to create a “scratch” (T0). Dermal fibroblasts (NHDFs) were then treated for 24 h with 0.1% *v*/*v* VPDS upon serum starvation conditions. Photos were taken at T0 and after 24 h with ZEN Microscopy Software (Zen lite 2012 blue edition, Zeiss, Vienna, Austria).

### 4.9. Collagenase Assay

The assay was based on spectrophotometric methods reported in the literature with some modifications for use in a microplate reader (150 μL total volume of the final reaction mixture) [55]. The assay was performed in a 50 mM tricine buffer with 10 mM calcium chloride and 400 mM sodium chloride, pH 7.5. Collagenase from *Clostridium histolyticum* (EC.3.4.24.3) was dissolved in a buffer at an initial concentration of 0.8 U/mL (0.1 U/mL final concentration). The synthetic substrate N-[3-(2-furyl) acryloyl]-Leu-Gly-Pro-Ala (FALGPA) was dissolved in a buffer at an initial concentration of 2 mM (final concentration 1 mM). The tested compounds were incubated with the enzyme in a buffer for 15 min. After the incubation the substrate is added. The absorbance was measured immediately after the addition of the substrate, at 345 nm and then every 1 min for 10 min using microplate reader (infinite 200 M Pro, Tecan, Männedorf, Switzerland). Ascorbic acid (AA) was used as a positive control.

### 4.10. Gene and Protein Expression Analysis

For the isolation of total RNA (400 ng), as well as for cDNA synthesis, the NucleoSpin RNA kit Macherey-Nagel, Germany and the PrimeScript RT reagent kit (Takara Bio, Kusatsu shi, Japan), were used, respectively. The qPCR method, as well as the process of gene analysis, was conducted as previously described. For the relative gene expression, the comparative threshold cycle method was used. GAPDH was used as normalization control.

Collagen and elastin expression was quantified by ELISA (Fine tests) in 450 μg/mL protein lysates, according to the manufacturer’s protocol.

### 4.11. Immunofluorescence Analysis

Human dermal fibroblasts at initial density of 10.000 cells/well and maintained in optimal growing conditions for 2 days. At day 1, particulate matters (ERMCZ100 PM10-like—PAHs; 1 μg/cm^2^) were added to the cells belonging from “Stress” groups and “Stress + VPDS” groups. After 1 h of contact the cells were irradiated with UVA (LED source, 365 nm, 3 J/cm^2^) in an irradiation chamber.

Immediately after the irradiation, the culture medium was renewed, the active ingredient added to the cells (0.1%) and left in contact for 24 h. The control group did not receive any treatment, except from the renewal of the culture medium. After 24 h of incubation, the cells were fixed on the plate and analyzed by in situ immunofluorescence detection, visualization and quantification of collagen-I, collagen-IIIa, and elastin signals.

### 4.12. Statistical Analysis

Results are expressed as the mean ± SEM of two different experiments. Statistical analysis between two individual groups was performed by Student’s *t*-test and one-way ANOVA (Dunnett’s multiple comparisons test). A *p* ≤ 0.05 was considered statistically significant. Statistical analysis and graphs were performed with Sigma Plot Software v.10 and GraphPad Prism 9 (GraphPad software Inc., San Diego, CA, USA).

## 5. Conclusions

The new raw material presents a novel, controlled way of dealing with antioxidants delivery to human skin for anti-aging reasons. It exhibits antioxidant and ECM protective effects when administered in skin fibroblasts (NHDFs). Treatment of NHDFs with the combinatorial delivery system for vine extract and propolis polyphenols promoted collagen end elastin synthesis and deposition under normal and stressed conditions. Therefore, this liposome–cyclodextrin encapsulated polyphenol system represents a novel bioactive and sustainable approach for cosmetics production with promising skin applications.

## 6. Patents

Patent WO/2023/222267 is based on the results of this publication.

## Figures and Tables

**Table 1:** Hydrodynamic diameter, polydispersity index, and ζ-potential of the system at day 1, and after 1 month at various temperatures.

Time	Conditions	z-Average Diameter (nm)	SD	PI	SD	ζ-pot (mV)	SD
T (0)	25 °C	444	63	0.455	0.059	−18	5.2
Day 30	6 °C	501	50	0.477	0.084	−16	3.0
Day 30	38 °C	821	199	0.616	0.105	−21	2.9
Day 30	25 °C	609	111	0.516	0.099	−22	3.2

**Table 2:** Evaluation of stability test of DPPH: Antioxidant activity (± SD) and TPCs (± SD) of the VPDS.

Time	Conditions	TPC (μg GA/mL)			DPPH (mM Trolox Equivalent)		
T(0)		709	±	22	5.23	±	2.36
Day 30	RT	640	±	38	4.75	±	2.20
	6 °C	535	±	44	4.96	±	2.16
	38 °C	550	±	31	4.59	±	1.63
	25 °C	502	±	28	4.73	±	1.67

## Data Availability

All study data available upon request.

## Abbreviations

AQP-3, Aquaporin-3; CD44, cluster of differentiation 44; DPPH, 2,2-Diphenyl-1-picrylhydrazyl; ECM, extracellular matrix; EGF, epidermal growth factor; GA, gallic acid; HA, hyaluronic acid; HAS-2, hyaluronan synthase-2; MTT, methylthiazolyldiphenyl-tetrazolium bromide; NHDFs, normal human diploid fibroblasts; ROS, reactive oxygen species; SOD, superoxide dismutase; TGF, transforming growth factor; TPC, total phenolic content; VEGF, vascular endothelial growth factor; VPDS, vine propolis delivery system.
